# Evaluation of Changes in the Motor Network Following BCI Therapy Based on Graph Theory Analysis

**DOI:** 10.3389/fnins.2018.00861

**Published:** 2018-11-27

**Authors:** Mohsen Mazrooyisebdani, Veena A. Nair, Po-Ling Loh, Alexander B. Remsik, Brittany M. Young, Brittany S. Moreno, Keith C. Dodd, Theresa J. Kang, Justin C. William, Vivek Prabhakaran

**Affiliations:** ^1^Department of Electrical and Computer Engineering, University of Wisconsin–Madison, Madison, WI, United States; ^2^Department of Radiology, University of Wisconsin–Madison, Madison, WI, United States; ^3^Department of Statistics, University of Wisconsin–Madison, Madison, WI, United States; ^4^Department of Kinesiology, University of Wisconsin–Madison, Madison, WI, United States; ^5^Neuroscience Training Program, University of Wisconsin–Madison, Madison, WI, United States; ^6^Medical Scientist Training Program, School of Medicine and Public Health, University of Wisconsin–Madison, Madison, WI, United States; ^7^Department of Biomedical Engineering, University of Wisconsin–Madison, Madison, WI, United States; ^8^Department of Neurological Surgery, University of Wisconsin–Madison, Madison, WI, United States

**Keywords:** BCI therapy, brain-computer interface, stroke recovery, graph theory, motor functional recovery, motor network

## Abstract

Despite the established effectiveness of the brain-computer interface (BCI) therapy during stroke rehabilitation (Song et al., 2014a, 2015; Young et al., 2014a,b,c, 2015; Remsik et al., 2016), little is understood about the connections between motor network reorganization and functional motor improvements. The aim of this study was to investigate changes in the network reorganization of the motor cortex during BCI therapy. Graph theoretical approaches are used on resting-state functional magnetic resonance imaging (fMRI) data acquired from stroke patients to evaluate these changes. Correlations between changes in graph measurements and behavioral measurements were also examined. Right hemisphere chronic stroke patients (average time from stroke onset = 38.23 months, standard deviation (SD) = 46.27 months, *n* = 13, 6 males, 10 right-handed) with upper-extremity motor deficits received interventional rehabilitation therapy using a closed-loop neurofeedback BCI device. Eyes-closed resting-state fMRI (rs-fMRI) scans, along with T-1 weighted anatomical scans on 3.0T MRI scanners were collected from these patients at four test points. Immediate therapeutic effects were investigated by comparing pre and post-therapy results. Results displayed that th average clustering coefficient of the motor network increased significantly from pre to post-therapy. Furthermore, increased regional centrality of ipsilesional primary motor area (*p* = 0.02) and decreases in regional centrality of contralesional thalamus (*p* = 0.05), basal ganglia (*p* = 0.05 in betweenness centrality analysis and *p* = 0.03 for degree centrality), and dentate nucleus (*p* = 0.03) were observed (uncorrected). These findings suggest an overall trend toward significance in terms of involvement of these regions. Increased centrality of primary motor area may indicate increased efficiency within its interactive network as an effect of BCI therapy. Notably, changes in centrality of the bilateral cerebellum regions have strong correlations with both clinical variables [the Action Research Arm Test (ARAT), and the Nine-Hole Peg Test (9-HPT)]

## Introduction

Eight lakhs Americans experience a stroke each year, a number that is predicted to rise by 22% by 2030 (Go et al., 2013). Recent medical advances have decreased stroke mortality rates (Go et al., 2013). However, the growing number of stroke survivors continue to struggle as their independence are notably diminished. These survivors often suffer from persistent functional deficits, resulting in billions of dollars of economic costs each year (Towfighi and Saver, 2011). Kelly-Hayes et al. (2003) shows that acquisition of a lasting motor impairment is one of the most prominent sources of such functional deficits, with up to 50% of survivors suffering from hemiparesis, and 26% requiring assistance with activities of daily living (ADLs) 6 months post-stroke. Consequently, this expanding population of stroke survivors increases the demand for effective stroke rehabilitation therapies and mechanistic break-down of stroke recovery.

The most critical time-frame for significant post-stroke recovery has been shown to occur within the first few months following stroke onset (Stinear and Byblow, 2014). During this period before plateauing around 6 months post-stroke (Wolf et al., 2006, 2010; Dromerick et al., 2009; Cramer and Nudo, 2010), spontaneous biological recovery (SBR) plays a major role in the complex process of motor recovery. spontaneous motor and cognitive recovery may no longer occur within the same manner as it is observed during SBR. Although patients in the chronic stages of stroke recovery retain the capability of neuroplasticity (Caria et al., 2011; Ang et al., 2015), traditional therapies have not been effective after 6 months post-stroke. As a result, chronic stroke survivors have fewer options for recovery.

In the absence of effective traditional rehabilitation therapy for chronic stroke survivors, novel therapeutic techniques show success in generating some functional motor recovery beyond traditional rehabilitation window (Cramer and Nudo, 2010; Ang et al., 2015; Irimia et al., 2016).

Brain-computer interface (BCI) therapy is being used in non-traditional therapies for stroke rehabilitation. An increasing number of studies indicate that with different neuro-rehabilitative BCI therapy strategies, both acute and chronic stroke patients can achieve significant changes in behavioral measures [such as the Action Research Arm Test (ARAT), and the Nine-Hole Peg Test (9-HPT)] of persistent upper extremity (UE) impairment (Young et al., 2014a,b; Irimia et al., 2016; Remsik et al., 2016). One such strategies that was applied in the ongoing clinical trial [(NCT02098265) interventional, non-invasive closed-loop electroencephalography (EEG) based BCI therapy for the restoration of distal UE motor function in stroke survivors Song et al., 2014a, 2015; Young et al., 2014a,b,c,d, 2015; Remsik et al., 2016] is to use electroencephalography (EEG) to detect neural activity. The signals from the EEG are translated into a video-game simulation which responses to user's neural patterns. The video game simulation provides real-time feedback which allows the user to observe and learn to modulate their brain activity. This method may stimulate neuroplastic changes and exploit any recovery potential that remains after a patient reaches a functional plateau with traditional therapies.

BCI therapies are designed to reward the consistent production of specific brain activity patterns relative to other patterns in the context of an intended task. While growing number of studies (Muralidharan et al., 2011; Song et al., 2014a, 2015; Young et al., 2014a,b,c,d, 2015; Irimia et al., 2016) have shown the effectiveness of BCI therapies in rehabilitating volitional movements in stroke survivors, little is known about the network reorganization patterns that occur in stroke patients by such therapies.

### Overview of this study

The aim of this study was to determine topological changes in the motor network of chronic stroke patients who participated in BCI therapy. Task-free (resting-state) fMRI was chosen to map brain network changes as it is easily acquired on all patients irrespective of the degree of impairment. In order to evaluate reorganization of the motor network, a pure data-driven methodology known as the graph theoretical analysis was applied. The graph theory has been recognized in recent years as a novel method to study functional networks of the brain (Bullmore and Sporns, 2009; Wang et al., 2010).

The fundamental basis of graph theory is to represent a network in terms of nodes (or vertices) and links (or edges) between pairs of nodes. This approach helps researchers to describe topologies of complex networks by quantifying properties of a network (Wang et al., 2010). When representing a large-scale brain networks in this way, nodes are usually defined as anatomical brain regions and links can be represented as functional connectivity (FC) between these nodes, in which FC is defined as the magnitude of temporal correlation of the activity of two brain regions (Boccaletti et al., 2006). Functional segregation and integration have been recognized as the two most important principles when considering networks in the human brain (Wang et al., 2010). Graph theoretical methods also enable researchers to evaluate hubs in a network (Wang et al., 2010). In a complex network, hubs have an essential importance in controlling over flowing information.

In this study, functional segregation and integration of the executive motor network was examined via clustering coefficient (measure of segregation) and shortest path lengths of the network (measures of the integration) (Bassett and Bullmore, 2006), and two measures of centralities (i.e., betweenness centrality and degree centrality) was used to evaluate alteration of hubs.

The main hypotheses in this study were:

- Gradual improvement in the ipsilesional primary sensorimotor cortex during the stroke recovery–potentially as a result of SBR–has been observed in recent longitudinal studies (Carey et al., 2002; Wang et al., 2010). An increase in the regional centrality of the ipsilesional primary sensorimotor following the administration of BCI therapy was hypothesized.
- Behavioral measurements (i.e., ARAT and 9-HPT) were predicted to be correlated with changes in the topology of the motor network. Specifically, it was hypothesized that changes in graph properties (regional centrality, etc.) will correlate with gains in motor function. Similar associations between regional centralities of the motor network and improvement in some clinical outcomes have been reported in spontaneous stroke recovery during the acute stroke stage (Wang et al., 2010). Also the association with improvement in the pattern of activity in fMRI data and improvement in some clinical variable during chronic stage has been observed previously (Carey et al., 2002; Gauthier et al., 2008; Richards et al., 2008).

## Materials and methods

### Recruitment methods, exclusion criteria, and ethic statement

Thirteen patients who suffer from persistent upper extremity motor impairment caused by ischemic or hemorrhagic stroke were enrolled for the BCI therapy. All of these subjects were recognized as proper for participation in this study by one or more physicians at the University of Wisconsin Hospital and Clinics. Patients with concurrent neurodegenerative disorders, such as dementia, or other neurological or psychiatric disorders, such as epilepsy, schizophrenia, or substance abuse, were excluded from this study. All subjects provided written informed consent. This study was approved by the Health Sciences Institutional Review Board of the University of Wisconsin–Madison. Participant characteristics are summarized in Table 1.

**Table 1:** Clinical and demographic data.

**ID**	**Infarcted hemisphere**	**Localization of infarct**	**ARAT affected hand Score**		**9-HPT Score**	
1	Right	Temporal, Frontal	3	3	29.31	21.06
2	Right	Occipital	57	57	27.5	22.99
3	Right	Temporal, Frontal	9	10	37.12	32.52
4	Right	Frontal	3	16	20.93	20.6
5	Right	Putamen	–	–	24.61	23.62
6	Right	Pons	27	40	30.51	28.00
7	Right	Cerebellum	57	57	26.48	21.79
8	Right	PLIC putamen	23	40	26.69	20.71
9	Right	Prefrontal, Midfrontal, Temporal	–	–	37.84	34.97
10	Right	Internal capsule, Thalamus	56	57	20.05	18.22
11	Right	Frontal, Parietal	7	7	19.46	18.62
12	Right	Frontaltemporal, Occipital	3	4	20.29	18.58
13	Right	Anterior temporal, Frontoparietal	0	2	26.77	24.25

### Randomization and study paradigm

All participants in this study were randomly assigned to one of two groups (BCI therapy group or crossover control group) using a permuted-block design accounting for gender, stroke chronicity, and severity of motor impairment. Those in the BCI therapy group immediately received interventional rehabilitation therapy using the BCI device with functional assessment and MRI scanning at four time points: before the start of BCI therapy (Pre therapy), at the midpoint of BCI therapy, upon completion of all BCI therapy (Post therapy), and 1 month following the last BCI therapy session. Those in the crossover control group completed three additional functional assessments and MRI scans during the control phase of the study and then crossed over to complete the same BCI therapy phase of the study as the first group. For more information about the study paradigm and details about interventions, please refer to Young et al. (2014a). Data analyzed in this paper is from the intervention phase for both groups and using only two time points: before therapy (or therapy baseline) and post-therapy. This is because several of our studies have shown the most significant gains following therapy at these time-points (Young et al., 2014b,d; Remsik et al., 2016).

### Functional assessments

Subjects' motor function of the impaired arm was assessed with behavioral objective measures. These measures included subjects' performance in the Action Research Arm Test (ARAT)–a standardized series of scored movements designed to evaluate upper extremity motor function in the domains of grip, grasp, strength, and gross movement (Carroll, 1965; Beebe and Lang, 2009; Young et al., 2014b), and the Nine-Hole Peg Test (9-HPT)–a timed task in which the subject attempts to first place the pegs in each of the 9 holes on a pegboard and then removes each peg using only one hand (Carroll, 1965; Young et al., 2014b). These scores were standardized as follows: scores for the ARAT were reported as the total points scored when using the impaired hand, and scores for 9-HPT were taken as an average of two timed trials using the impaired hand (Young et al., 2014b).

At each of the visits for behavioral evaluation, anatomical and functional MRI scans were also obtained for each subject.

### Image acquisition and processing

MRI data were collected on 3 Tesla GE MR750 scanners equipped with high-speed gradients (Sigma GE Healthcare, Milwaukee, Wisconsin) using an 8-channel head coil. In order to minimize head movements, padding was used around each subject's head. Ten minutes resting-state (R-s) fMRI data were collected using a T2^*^-weighted gradient-echo planar imaging (EPI) pulse sequence sensitive to BOLD contrast. Technical parameters used to acquire these EPI scans were as follows: field of view 224 mm, matrix 64 × 64, TR 2600 ms, TE 22 ms, flip angle 60°, and 40 axial plane slices of 3.5 mm thickness with 3.5 mm spacing between slices. A T1-weighted high-resolution anatomical image was also obtained for each subject using a BRAVO FSPGR pulse sequence. Technical parameters used to acquire these scans are as follows: field of view 256 mm, matrix 256 × 256, TR 8.16 ms, TE 3.18 ms, flip angle 12°, and 156 axial plane slices of 1 mm thickness with 1 mm spacing between slices.

R-s fMRI data were processed using the AFNI package (Cox, 1996). Images were despiked, slice time-corrected, motion-corrected, aligned with the anatomical scan, normalized to MNI space, re-sampled to 3.5 mm, and spatially smoothed with a 4 mm FWHM Gaussian kernel. Motion censoring (per TR motion > 1 mm or 1°), nuisance regression, and bandpass filtering (0.009–0.08 Hz) were performed simultaneously in one regression model. Nuisance signals that were regressed out included six motion estimates and their temporal derivatives, the voxel-wise locally averaged white matter signal, and the cerebrospinal fluid signal. Global signal regression was omitted due to the controversial position associated with it in the literature (Murphy and Fox, 2017).

### Graph construction

**Figure 1:**
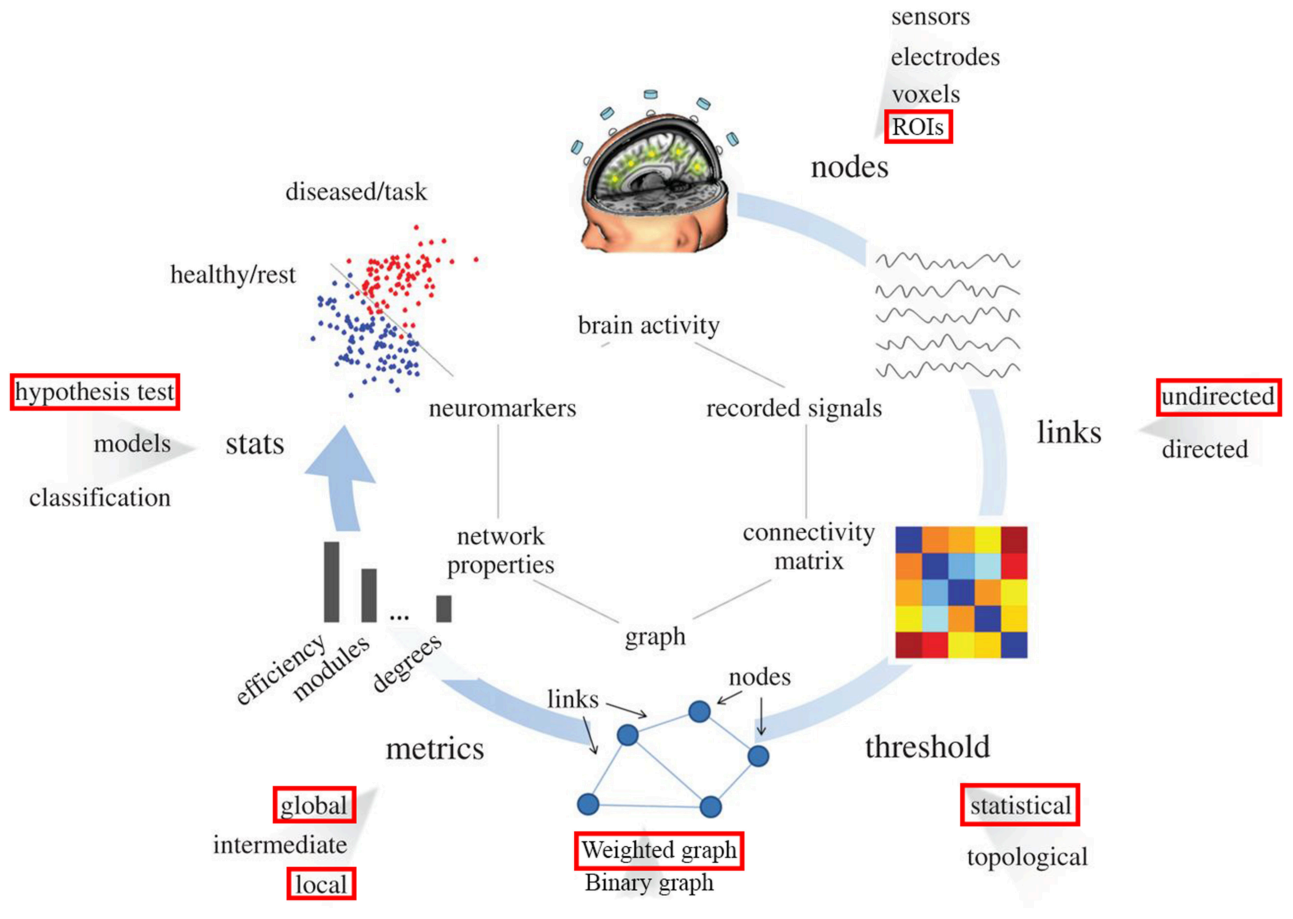
Pipeline for the graph theory analysis applied on functional brain network. Red rectangulars specify the submethodology used in this study at each step. Nodes correspond to specific region in the brain (predifined ROI in our study). Links are estimated by measuring the FC between different regions in the brain (undirected links); connectivity matrix would be constructed using this information. By means of filtering procedures, based on thresholds, only the most important links constitute the brain graph. The topology of the brain graph is quantified by different graph metrics that can be represented as numbers. These graph indices can be input to statistical analysis in order to look for significant differences between populations/conditions (e.g., red points correspond to brain graph indices of diseased patients or tasks, blue points stand for healthy subjects).

Figure 1 illustrates the standard procedure of graph theory analysis applied on f-MRI data that has been well-stablished and used in many studies (Humphries et al., 2006; Achard and Bullmore, 2007; He et al., 2008; Bullmore and Sporns, 2009, 2012; Meunier et al., 2009; Alexander-Bloch et al., 2010; Van Wijk et al., 2010; Wang et al., 2010; Bernhardt et al., 2011; De Vico Fallani et al., 2014; Song et al., 2014b). Reign of interest (ROI) from the network under investigation is first identified. These ROIs would be nodes in the graph. Then the correlation matrix (or functional connectivity (FC) matrix) between these ROIs is acquired using temporal correlations among all ROIs. Next, the proportional thresholding is applied to exclude weak or irrelevant FCs from the analysis of the graph. A threshold value in the context of proportional thresholding (known as network sparsity) is defined as the number of correlations that is considered as connections in the final graph divided by number of all possible correlations exist in the correlation matrix (Latora and Marchiori, 2001; Achard et al., 2012). After proportional thresholding and excluding weak FCs, each remaining FC is identified as a link (or edge) between its associated ROIs and the graph is constructed. From this graph, topological properties of the network under investigation can be evaluated.

Optimally thresholding correlation matrix to only include important FCs is critical in this methodology. Having too few FCs may obscure group differences, whereas too many FCs may lead to a random graph structure (Humphries et al., 2006). However, applying this method on a brain network model has a potential to move the graphical model away from the actual network that it represents. In the section Preserving graph connectedness and network thresholding, this limitation of the thresholding is explained and a technique (the Maximum Spanning Tree, MST) to circumvent this potential limitation is introduced.

In the following subsections, the criterion for choosing ROIs in our study is explained, and the proportional thresholding is discussed in more details.

### Regions of interest in executive motor network

Twenty-one anatomical ROIs associated with the motor execution network were defined by creating 5 mm diameter spheres around coordinates for regions in the motor network previously defined by Wang et al. (2010) (Table 2). One ROI (located in the right ventrolateral premotor cortex) was excluded due to overlap with subject's stroke lesions. The 20 ROIs include the primary motor cortex, bilateral superior parietal lobule, bilateral basal ganglia, bilateral thalamus, anterior inferior cerebellum, postcentral gyrus, and dentate nucleus (Wang et al., 2010). These ROIs were used to derive Pearson's R correlation coefficient matrices from each subject's r-s fMRI, using AFNI's doROICorrMat command. Fisher z transform was then applied on R correlations across each patient and used z-score correlation matrices in further analysis (Since hypotheses about the significance of the population correlation wanted to be evaluated, Fisher z-score was more proper than r-correlation value). In this study, the alteration in the magnitude of the functional connections was tended to be evaluated; hence, absolute values of these matrices were used in all analyses.

**Table 2:** Regions of interest for the motor network.

**ID**	**Region**	**Abbreviation**	**Side**	**MNI coordinate**		
				***x***	***y***	***z***
1	Superior cerebellum	SCb	R	16	−59	−21
2	Primary motor cortex	M1	L	−38	−22	56
3	Primary motor cortex	M1	R	38	−22	56
4	Thalamus	Th	L	−10	−20	11
5	Superior parietal lobule	SPL	L	−22	−62	54
6	Supplementary motor area	SMA	L	−5	−4	57
7	Supplementary motor area	SMA	R	5	−4	57
8	Dorsolateral premotor cortex	PMd	R	28	−10	54
9	Ventrolateral premotor cortex	PMv	L	−49	−1	38
10	Superior cerebellum	SCb	L	−25	−56	−21
11	Superior parietal lobule	SPL	R	16	−66	57
12	Dentate nucleus	DN	R	19	−55	−39
13	Anterior inferior cerebellum	AICb	L	−22	−45	−49
14	Anterior inferior cerebellum	AICb	R	16	−45	−49
15	Postcentral gyrus	PCG	R	37	−34	53
16	Dorsolateral premotor cortex	PMd	L	−22	−13	57
17	Basal ganglia	BG	R	22	−2	12
18	Basal ganglia	BG	L	−25	−14	8
19	Thalamus	Th	R	7	−20	11
20	Dentate nucleus	DN	L	−28	−55	−43

*MNI, Montreal Neurological Institute; R, Right; L, Left*.

### Preserving graph connectedness and network thresholding

As it is described earlier in this section, applying thresholding without any consideration for the reality of the circulation of information in the network has some potential issues. Thresholding raises two critical issues; (1) It may lead the final graph to be disconnected–in which a region that is part of the brain network will be left without any connection to any other region in the graph, (2) In addition, there is no comprehensive agreement in the field on the cutoff value above which correlations should be considered as edges.

To address the first issue a growing number of studies have used the maximum spanning tree (MST) method (Alexander-Bloch et al., 2010; Achard et al., 2012; Song et al., 2014b; Iyer et al., 2018). An MST is a weighted spanning tree that would serve as a backbone for the main graph. In this method, to calculate the existing tree in the graph with the maximum weights, N-1 FCs is chosen by the prime algorithm to connect all N nodes of the network together.

As for the second issue, analysis of the graph in the whole-network level (such as evaluation of the shortest path length, clustering coefficient, small-worldness, etc.) has been done in various numbers of threshold values in almost all previous studies (Loui et al., 2012; Rutter et al., 2013; Vaessen et al., 2014; He et al., 2017). This was to capture a proper and complete understanding of the network topology.

For regional properties of the network (e.g., centrality, or local efficiency) however, there is still a debate about the proper threshold value. For instance, Bullmore and his colleagues (Bullmore and Sporns, 2012) believe that each node in a graph conforms to the profile of its realistic brain region only in small threshold values not more than 16% (same thresholding criteria has been used in Meunier et al., 2009). Another example is Iyer et al. (2018) in which the author used 6% as the threshold value. However, in the growing numbers of studies researchers have used all significant correlations to construct the brain graphical model (Alexander-Bloch et al., 2010; Wang et al., 2010; Achard et al., 2012; Song et al., 2014b).

In this study, all significant correlations were used to generate the graph of each patient's brain in order to analysis of regional properties. From each patient's connectivity matrix, *z*-values > 1.96 (two-tailed significant value for z-score) were used as the threshold to identify percentage of correlations that are above this threshold, i.e., the ratio of significant connections to all the possible connections were calculated (Supplementary Figure 1). By this approach, it has been found that the minimum sparsity was more than 42%. Hence, the sparsity threshold of 42% was used to convert connectivity matrices into weighted networks.

In summary, after applying the MST and extracting the backbone, any other FCs identified as a connection in the thresholding step are added to this tree to get weighted undirected connection matrices that represent a sparse, connected, and biologically meaningful graph for each patient (Song et al., 2014b).

While most of brain network studies have investigated the brain's topology by analyzing binaries graph (in which every edge in the network has an equal weight of 1), here alteration in the executive motor network was evaluated by a weighted network analysis approach, in which every edge in the network has a weight equal to its equivalent FC in the connectivity matrix, and hence the network would contain more information about the actual brain circuity.

### Graph measurements

#### Weighted clustering coefficient and weighted shortest path length

The clustering coefficient (C) is a measure of the degree to which nodes in a graph tend to cluster together (Watts and Strogatz, 1998). For an undirected weighted graph, the clustering coefficient of a node *i* (*ci*) is defined as follows:

$$\begin{array}{l} C_{i}=\frac{1}{S_{i}\left(K_{i}-1\right)}\underset{\left(j,k\right)}{\sum }W_{ij}+W_{ik} \end{array}$$

Here, S_i_ is the strength of the node *i* (defined as sum of the FC between node *i* and other regions), W_ij_ is the FC between node *i* and node *j*, and K_i_ is the number of edges connected to the node *I*. The sum over *(j,k)* carries out sum of weights for any two pairs of *j* and *k* connected to the node *i* (Wang et al., 2010; Bernhardt et al., 2011). The clustering coefficient over all nodes in a network is then defined as:

$$\begin{array}{l} C=\frac{1}{N}\overset{N}{\underset{i=1}{\sum }}c_{i} \end{array}$$

The characteristic path length (L) reflects the level of global integration in the network. A shortest path between two nodes A and B is the path between A and B with the smallest number of edges. The characteristic path length *l*_*i*_ of a node *i* is defined as Watts and Strogatz (1998):

$$\begin{array}{l} l_{i}=\;\frac{1}{N-1}\underset{i\neq j}{\sum }\min\left\{I_{ij}\right\} \end{array}$$

Where min{*Iij*} is the shortest path length between the i^th^ and j^th^ nodes. The characteristic path length L of a network is then defined as the mean of characteristic path lengths over all nodes in the network:

$$\begin{array}{l} L=\frac{1}{N}\overset{N}{\underset{i=1}{\sum }}l_{i} \end{array}$$

#### Regional centrality measurements

In network analysis, indicators of centrality identify the most important nodes within a graph (Brandes, 2001). In the present study, each node's importance in the network was evaluated using degree centrality and betweenness centrality.

Degree centrality (DC) counts the number of neighbors of each node. In this context, a node with higher degree centrality, would have more FCs with other parts of the network and hence is more involved in the network communication.

Betweenness centrality (BC) captures the influence that one node has over the flow of information between all other nodes in the network. The betweenness centrality of a node *v* is calculated as follows (Brandes, 2001):

$$\begin{array}{l} BC\left(v\right)=\underset{S\neq v\neq t}{\sum }\frac{\sigma_{st}\left(v\right)}{\sigma_{st}} \end{array}$$

Where σ_st_ is the total number of shortest paths from node s to node t and σ_st_(*v*) is the number of shortest paths from node s to node t that passes through node v. A node with high centrality is considered to be a hub in the network. Since this summation scales with the number of pairs of nodes, the quantity is rescaled and normalized by the average of BC over all nodes (Wang et al., 2010).

In this work, all graph measurements were calculated by using the Brain Connectivity Toolbox (2016) in MATLAB R2015.

### Statistical analysis

All tests between two time-points were assessed using non-parametric Wilcoxon signed-rank test. For all statistical tests α was set to 0.05 and then for each family of tests (i.e., tests of betweenness centrality, degree centrality, and correlations), correction for multiple comparisons were performed separately using false discovery rate (FDR) (Benjamini and Hochberg, 1995). All *p*-values reported in this study are unadjusted *p*-values (i.e., *p*-values are not FDR adjusted *p*-values, also known as q-values) and after FDR correction, any significant test was reported and marked with asterisk in the figures and tables. Tests with *p*-values < 0.07 were also considered trend toward significance and marked with plus in figures and tables.

## Results

### Participant characteristics and behavioral outcomes

**Figure 2:**
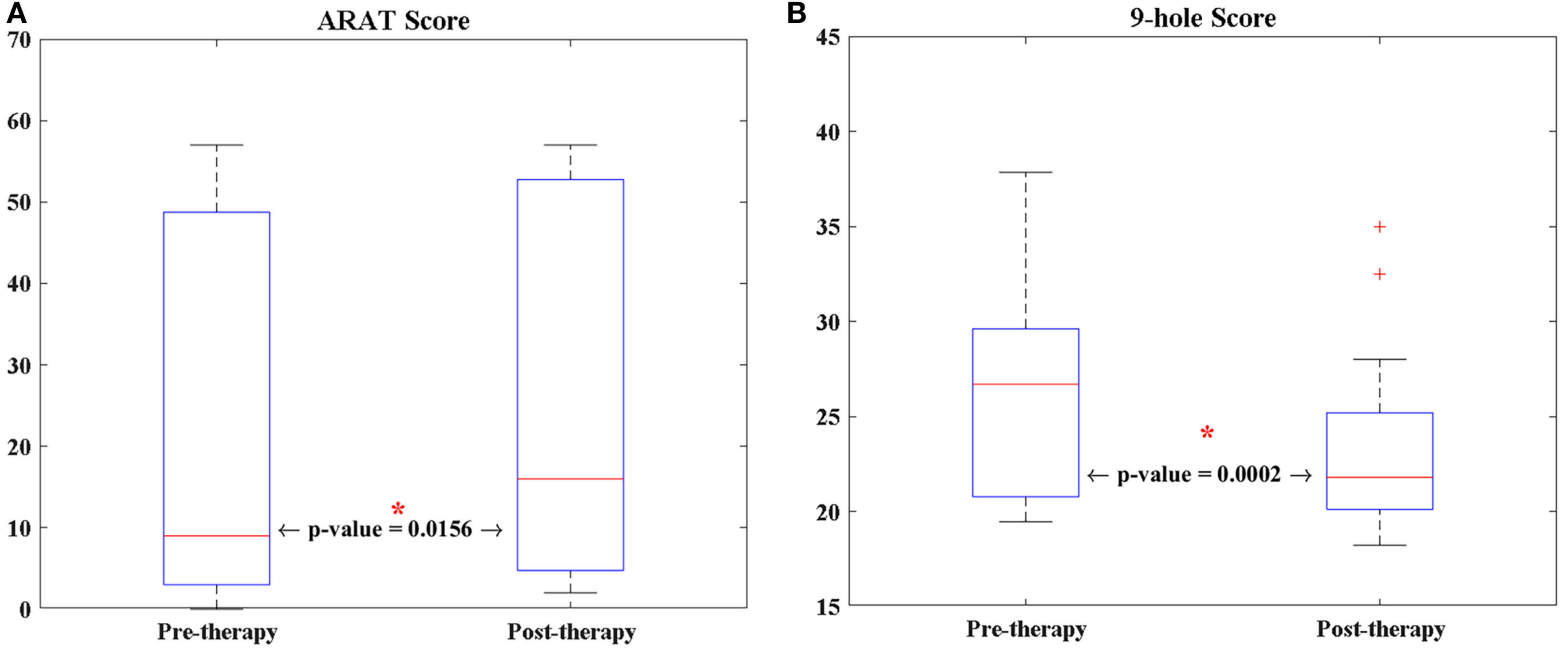
The longitudinal changes of patients' performance in **(A)** ARAT, and **(B)** 9-HPT scores analyzed via Wilcoxon signed-rank test. 9-HPT, Nine-Hole Peg Test; ARAT, Action Research Arm Test. *Indicates that *p*-value is significant (*p* < 0.05).

The average age of the 13 participants in this study was 64.92 years (*SD* = 12.19 years), and the average time from stroke onset was 38.23 months (*SD* = 46.28 months). Of the 13 patients, two patients were unable to perform the ARAT (Table 1). For the other participants, a Wilcoxon sign rank test was performed on each of the behavioral scores (i.e., 9-HPT, and ARAT) (Figure 2). Compared to pre-therapy, both the 9-HPT and ARAT scores demonstrated significant recovery (*p* = 0.0156 for ARAT and *p* = 0.0002 for 9-HPT).

### Adjacency matrices

**Figure 3:**
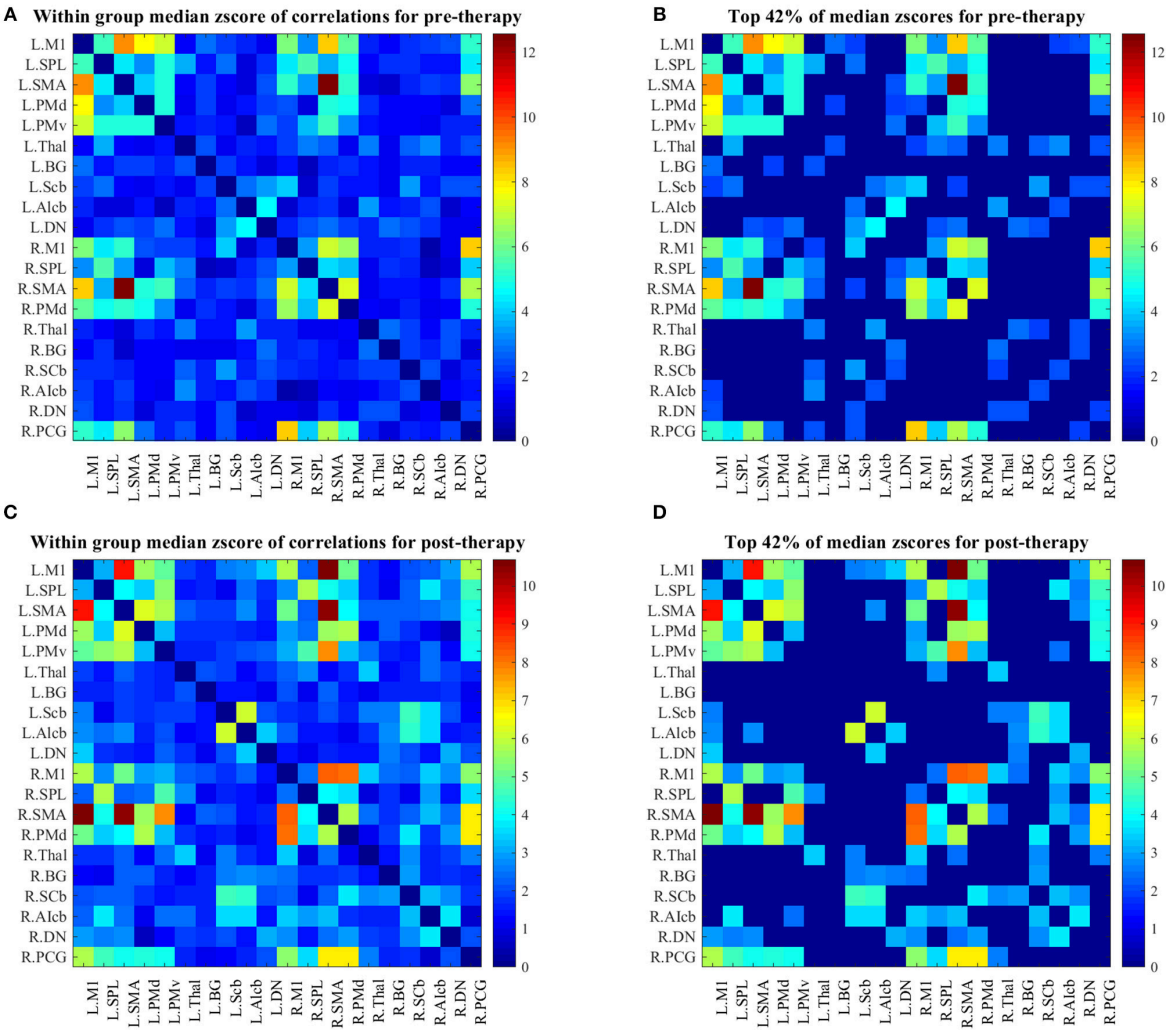
**(A)** Median z-score of r-correlation matrices in pre-therapy. **(B)** Median z-score of r-correlation matrices for pre-therapy at threshold value = 42%. **(C)** Median z-score of r-correlation matrices in post-therapy. **(D)** Median z-score of r-correlation matrices for post-therapy at threshold value = 42%. R = Right, L = Left. See Table 2 for the abbreviations of the regions. Note that the correlation matrices presented only serve as a visual representation, and are not corrected for multiple comparisons.

Changes in group-level FCs between two scans were evaluated by median–a more robust measure of central tendency compared to mean–of each group's z-score connectivity matrices. As depicted in Figure 3, patients showed higher FCs among the contralesional subcortical regions (thalamus and basal ganglia) and other contralesional sensorimotor regions before therapy (Figure 3B). The median metric after therapy showed a decrease in the FCs of these regions while ipsilesional sensorimotor and subcortical regions of the motor network showed increased their FC with other parts of the network (this is clear from comparing the entries in the bottom right of the matrix in Figure 3D with same entries of the matrix in Figure 3B).

### Global network parameters

**Figure 4:**
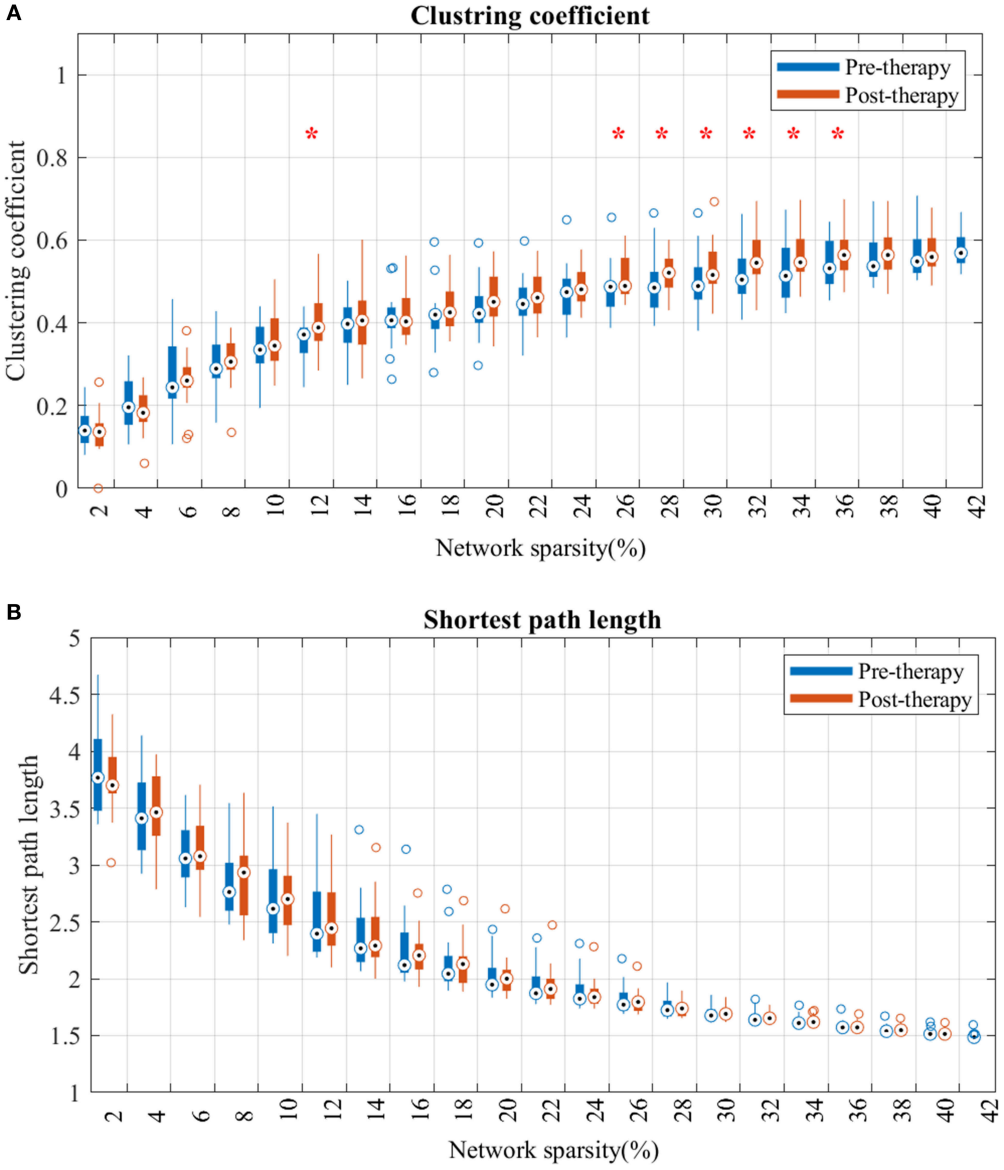
Changes in clustering coefficient **(A)** and average shortest path length **(B)** from pre-therapy (Blue) to post-therapy (red) across range of networks' sparsity. Vertical lines denote the standard deviation of each group. Statistical analyses were carried out using Wilcoxon signed-rank test. *Indicates significant after correction for multiple comparison.

Analysis of the shortest path length of the brain network showed no significant differences at any sparsity level over the study period (Figure 4B).

For the clustering coefficient (Figure 4A), mean clustering coefficient at post-therapy showed significant increase, comparted to pre-therapy, across several threshold values. Specifically, the network consisting of strongest FCs (sparsity lower than 12% in Figure 4B) showed no significant difference from pre to post. However, after including more mild edges (network sparsity between 12 and 36%), clustering coefficients in post-therapy gradually increased, and the gap between each time-point's distribution broadened as the sparsity increased.

### Local centrality parameters

**Figure 5:**
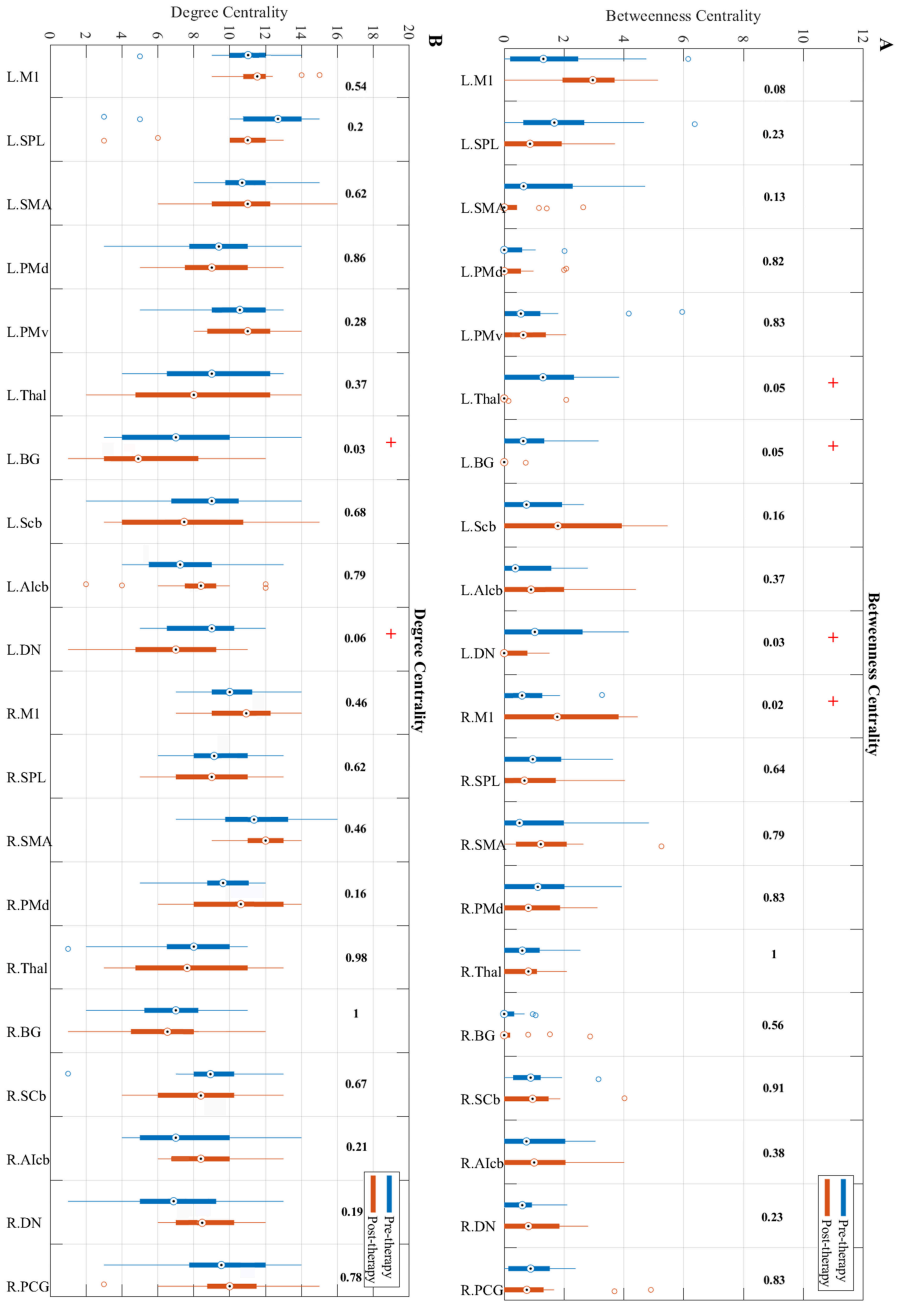
Changes in betweenness centrality **(A)** and degree centrality **(B)** measures from pre-therapy (Blue) to post-therapy (Red) across all regions in the network calculated at a density level of 42% analyzed via Wilcoxon signed-rank test. R, Right, L, Left. See Table 2 for the abbreviations of the regions. + trend toward significance (i.e., raw *p*-value < 0.07). *P*-values are round up with 2 integers in order to be shown in the figure.

Betweenness centrality showed a trend toward significant increase from pre to post-therapy (Figure 5A) in the ipsilesional primary motor cortex (*p* = 0.0201). While the contralesional dentate nucleus, basal ganglia, and the thalamus in post-therapy showed a trend toward significant decrease in BC compared to pre-therapy (*p* = 0.0324, *p* = 0.0502, and *p* = 0.0537, respectively).

Changes in the degree centrality of the motor network over the study period were investigated (Figure 5B). Results indicate that compared to pre-therapy, the degree centrality of the contralesional dentate nucleus (*p* = 0.0593) and basal ganglia (*p* = 0.0334) decreased over the study period.

### Behavioral correlations with changes in network parameters

**Figure 6:**
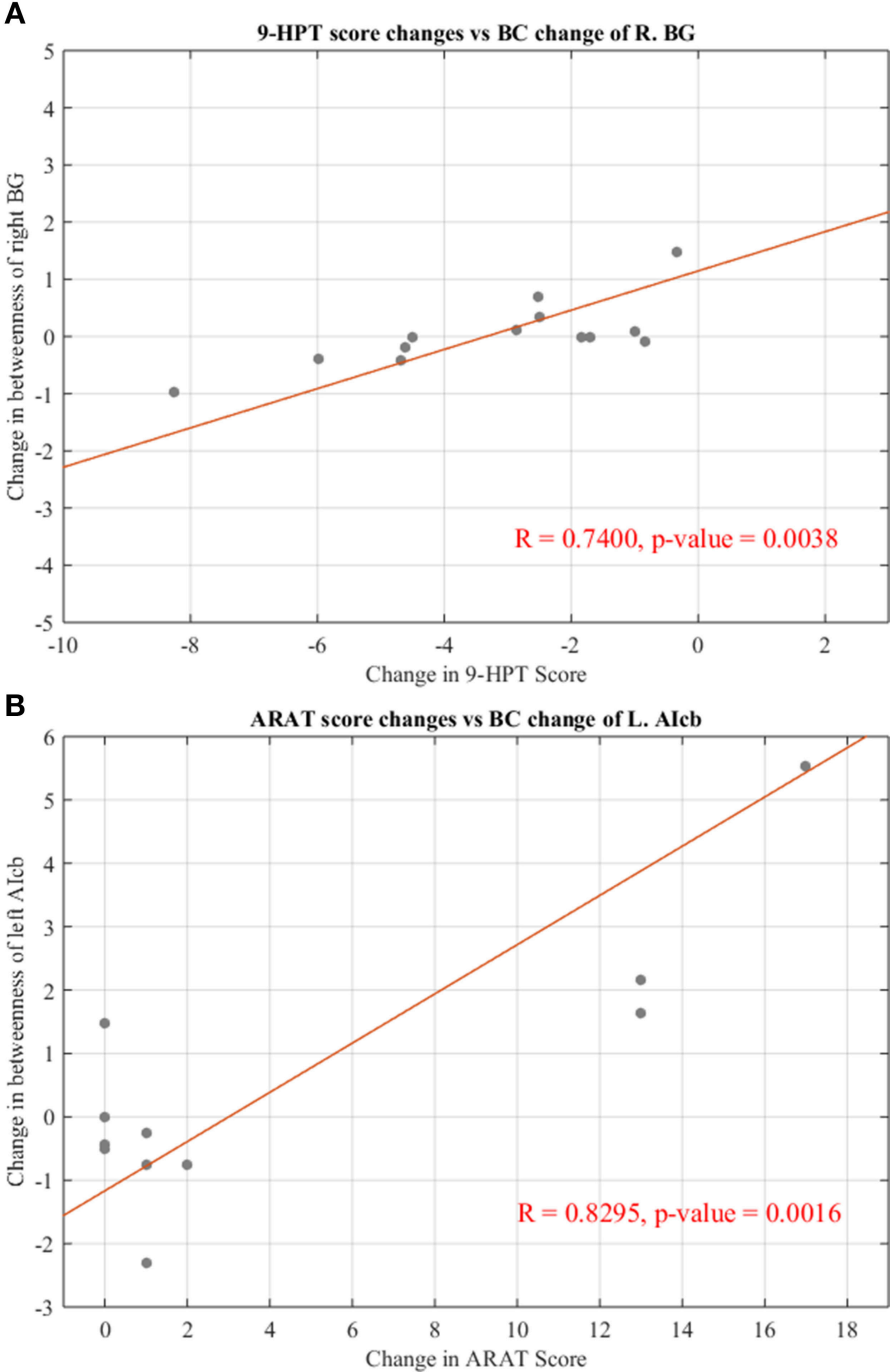
Significant correlations between changes in regional centralities and changes in behavioral measures. **(A)** Relationship between changes in BC measure of right basal ganglia and individual changes in 9-HPT score. **(B)** Relationship between changes in BC measure of left anterior inferior cerebellum and individual changes in ARAT score. Red line representing the slope of correlation between measurements. 9-HPT, Nine-Hole Peg Test; ARAT, Action Research Arm Test; R, Right; L, Left. See Table 2 for the abbreviations of the regions.

To examine the behavioral implications of the changes in graph theoretical measures, the linear associations between changes in network parameters and actual recovery reflected in the behavioral assessments were examined. A summary of Pearson's correlations between changes in outcome measures (ARAT and 9-HPT scores) and changes in network parameters found to be significant or showing a trend toward significance after FDR correction is presented in Table 3. The majority of these relationships involved the bilateral cerebellum. Changes in centrality of the contralesional anterior inferior cerebellum were highly correlated with both objective measurements (ARAT and 9-HPT). Figure 6 presents graphs of the relationships that were found to be significant.

**Table 3:** Correlation analysis between centrality changes and behavioral changes from pre- to post-BCI therapy assessments.

**Behavioral measure**	**Graph measure**	**Pearson *R*-value**	***P*-value**
ARAT	L.AIcb (BC)	0.8295	*0.0016
ARAT	R.Scb (BC)	−0.6832	^+^0.0205
ARAT	R.BG (BC)	0.6458	^+^0.0318
ARAT	L.AIcb (DC)	0.6022	^+^0.0499
9-HPT	R.BG (BC)	0.7400	*0.0038
9-HPT	R.DN (BC)	0.5720	^+^0.0411
9-HPT	L.AIcb (DC)	−0.5589	^+^0.0471
9-HPT	R.BG (DC)	0.6237	^+^0.0227

ARAT, Action Research Arm Test; 9-HPT, 9-Hole Peg Test; R, Right; L, Left; ^*^Significant p-value after correction for multiple comparison with FDR, ^+^ trend toward significant (i.e., p < 0.07). See Table 2 for the abbreviations of the regions.

## Discussion

### Effectiveness of rs-fMRI in evaluation of recovery in stroke

This study demonstrates the effectiveness of rs-fMRI using graph theoretical methods to capture brain changes during the stroke recovery following rehabilitative therapy. rs-fMRI requires about 10 min for image acquisition without any exogenous task demands on the subject. This method is particularly well-suited for stroke patients, who often suffer from motor impairment and hence may not be capable of doing specific tasks during MR scanning.

### Impact of BCI-based stroke rehabilitation on FC among regions of the motor network

This study shows that during the course of BCI therapy, the motor network strengthens its FC among different regions mostly in the ipsilesional part of the network. A similar study (Wang et al., 2010) in patients with subcortical infarcts in acute stage of recovery found significantly decreased FC involving the contralesional subcortical structures (such as the thalamus) during the recovery. Findings in the study highlights a similar pattern of decreasing median of FC in these regions (Figure 3, z-score connection matrices of left thalamus for pre-therapy Figure 3A compared to post-therapy Figure 3C). From this result, it seems that during the period of therapy, ipsilesional cortical and subcortical regions in the network have strengthened their FCs with other parts of the network.

### Graph theory as a tool to evaluate stroke recovery

Several studies have shown changes in the brain activation and functional connectivity following BCI therapy (Young et al., 2014a,b). The focus in this study was on investigating brain reorganization using network analysis methods. Specifically, graph theoretical methods were used to capture topological properties associated with therapy over time. Previous study (Wang et al., 2010) have used this mathematical method to determine changes in patients who were in the acute stage of stroke, when abnormal changes are more observable. Here, this method has been used to identify abnormal changes in chronic stroke patients with average time since stroke onset of 38.23 months. Results of this study demonstrate the efficacy of this method in detecting brain network changes in stroke patients over time following rehabilitative therapy.

### Effect of BCI-based therapy on the large-scale motor network

Changes in the topology of the motor network has been determined on a larger scale by evaluating the average clustering coefficients and the average shortest path lengths across all regions in the network. Results highlight that during the course of therapy, the clustering coefficient of the network increases significantly across different network sparsities (Figure 4). The higher clustering coefficient suggests that the brain follows principles of efficient network structures (Watts and Strogatz, 1998). Therefore, BCI therapy might help the motor network to facilitate more enhanced communication between communities of nodes (i.e., nodes sharing similar neighbors), resulting in faster transmission of information between brain regions.

### Alteration in regional centrality

Alterations in the importance of different regions in the motor network have been investigated in our study. The word “importance” has different meaning in different contexts, leading to different definitions of centrality (Borgatti, 2005). The importance of regions in facilitating information transfer within the network were evaluated using two different forms of centralities. Degree centrality, in the group of radial centralities (Borgatti and Everett, 2006), computes the number of edges connected to each node. This definition of centrality is particularly attractive, since a change in degree centrality is associated with a decrease or increase in the number of significant FCs of that node. A trend toward significant Decrease in degree centrality of the contralesional basal ganglia (*p* = 0.03) were observed in our study, similar pattern was observed in Wang et al. (2010). Also, a trend toward significant decrease (*p* = 0.06) has been found in the degree centrality on contralesional dentate nucleus.

This study also investigated the hub properties of nodes from the viewpoint of betweenness centrality. Betweenness centrality is a measure of the functional importance of a node in terms of being a bridge for information processing. In this context, most of the information flowing in the network passes through a node with high BC. Results showed a trend toward significant increased BC in the ipsilesional primary motor cortex (*p* = 0.02), which is similar to other studies (Wang et al., 2010), (Dong et al., 2007). Also, a trend toward significant decrease has been found in BC of contralesional subcortical regions (e.g., thalamus and basal ganglia). The decrease of BC in the contralesional dentate nucleus (*p* = 0.03) seen in our study was not observed in Wang et al. (2010). This may be due to the differences in study samples, with chronicity and stroke location in the patients varying between the two studies.

These findings suggest an increase in the role of ipsilesional primary motor area as a hub during the period of therapy. The increased important of ipsilesional primary motor areas may instigate the gradual recovery of contralesional affected hand in terms of contralateral motor control. Also it suggests a decrease in the role of the contralesional subcortical and cerebellum regions following therapy. One possible explanation for these findings might be that the recovery of overall brain connectivity in the ipsilesional subcortical and cerebellum regions. In other words, the connections going through the ipsilesional subcortical and cerebellum regions become stronger as a result of the therapy. This recovery might lead to a decreased role for the contralesional subcortical and cerebellar regions in transferring information within the motor network.

### Correlations between brain network changes and behavioral outcomes

Significant correlations between changes in centrality measures and changes in behavioral outcome measures are consistent with the view that the motor network changes with BCI therapy to facilitate information transfer between key regions in the motor network. Significant positive correlations between centrality of specific regions (e.g., anterior inferior cerebellum, and basal ganglia) and performance on the ARAT suggest that behavioral performance improves as the centrality of these regions increases. Similarly, significant negative correlations between centrality and 9HPT suggest that as centrality increases, processing time is reduced (i.e., time taken to perform the 9HPT is decreased). Interestingly, results showed similar correlations between centrality of the bilateral cerebellar regions and behavioral performance to that reported by Wang et al. (2010), in which the authors used the same ROIs. Also results from Dong et al. (2007) show reorganization of adaptive activity within the primary motor cortex and the cerebellum is in relation to relevant behavioral changes of patients with the upper extremity.

Cerebellar activity is solely associated with ipsilateral motor actions (Shibasaki et al., 1993; Allen et al., 1997). Addition studies have displayed that increased contralesional cerebellar activity is linked with the restoration of motor function (Chollet et al., 1991; Jaillard et al., 2005). Small et al. (2002) study further indicated that the larger the contralesional cerebellar activation, the better the recovery is Small et al. (2002). The results from this research study also mimic this trend.

However, it is common in individuals with upper-extremity motor impairments to overuse the unaffected arm more which may result in increases of centrality of the ipsilesional cerebellum. The negative correlation found between the centrality of ipsilesional superior cerebllum and ARAT performance—and ipsilesional basal ganglia with 9HPT performance—suggests that the recovery is enhanced by reducing over-recruitment of the contralateral extremity.

Overall these brain changes in subcortical structures (such as basal ganglia) and cerebellum and its interaction with cortical regions as well as the brain-behavioral correlations are consistent with these brain structures' involvements in movement related functions (i.e., basal ganglia has been implicated in functions including control of voluntary movements, procedural learning and the cerebellum contributes to functions such as coordination, precision, and timing of movements). However, given the small sample size and the fact that some of subjects were showing floor effects, these correlational results must be considered exploratory and interpreted carefully.

### Limitations and methodological considerations

This study had a limited sample size, given that we chose to focus on a relatively homogenous group of stroke patients, with all patients having right-sided lesions and being in a chronic stage. We thus eliminated the confounding effect of lesion hemispheres by choosing only right hemisphere patients. However, the localization of the infarct in the sample size is still heterogeneous within the right hemisphere. Therefore, results of this study should be interpreted with cautious. Future studies should be done with larger sample size and more homogenous infarct.

The rsFCs within the motor regions were constructed using the seed regions reported in the work of Wang et al. (2010), which studied spontaneous recovery in stroke patients. A large number of studies report slightly varying coordinates for the motor network; however, given that the Wang et al. seed regions cover crucial regions of the motor network, it was decided to construct RSFC matrices using these regions. By focusing on a within-groups analysis, effects of other confounding variables such as age, gender, and stroke severity were mitigated. Also, attempts to reduce false positives in results were made by applying the FDR correction and reported only those results that survived the corrected *p*-value. However, given the small sample size and the rehabilitative focus of the study, we have also reported results showing a trend toward significance, since these results, although statistically not significant, may have practical implications.

The findings of this study showcase effective theoretical approaches that may be further optimized in designing neurofeedback devices and paradigms for stroke recovery. These methods are also particularly useful when used to discern brain activity patterns for training and conditioning purposes. A review performed by Dimyan and Cohen (2011), determined that increased ipsilesional lateralization may be more optimal for motor recovery by its association with spontaneous recovery (Dimyan and Cohen, 2011). The conclusions made by Dimyan and Cohen (2011) are consistent with the possibility that is a diversity in neuronal pattering/organization that facilitate more effective recoveries following stroke. Furthermore, these progressive patterns may be modulated with interventional therapeutic technologies in ways that are not evicted by spontaneous recovery.

## Conclusions

This study provides a graph theoretical approach toward investigating brain changes following BCI therapy in chronic right hemisphere stroke patients with upper extremity motor impairments. Results showed that improvement in ipsilesional brain connectivity in the motor network can be observed concurrently with a period of training using a BCI device, and that these changes might be correlated with improved in behavioral outcomes. Due to small sample size and hetorogenous localization of the infarct in the sample size, these results should be interpreted with cautious and further studies will be needed with larger sample size to follow up on these findings. This study sheds light on the underlying mechanisms of recovery following BCI therapy, and may contribute toward developing more patient-specific BCI therapy protocols to facilitate recovery in chronic stroke patients.

## Author contributions

MM was involved in data collection, data analysis, interpreting results, and writing of the manuscript. VN contributed to data collection, manuscript writing, and intellectual content. P-LL contributed to editing the manuscript and intellectual content. AR, BM, and BY were involved in data collection and editing the manuscript. KD was involved in data collection. TK was involved in the recruitment of study participants. JW and VP are co-PIs and were involved in study conception, design, manuscript editing, intellectual content, and supervision for all aspects of the study.

### Conflict of interest statement

The authors declare that the research was conducted in the absence of any commercial or financial relationships that could be construed as a potential conflict of interest.

## Supplementary material

Supplementary Figure 1 Percentage of significant FCs for each patient in each session. Percentage of significant is defined as the number of significant FCs divided by the number of all possible FCs in the network.
