# Supplementary figures and images for: Evaluation of Changes in the Motor Network Following BCI Therapy Based on Graph Theory Analysis
Source: Front Neurosci. 2018 Nov 27;12:861. doi: 10.3389/fnins.2018.00861 (PMC6277805; doi:10.3389/fnins.2018.00861)

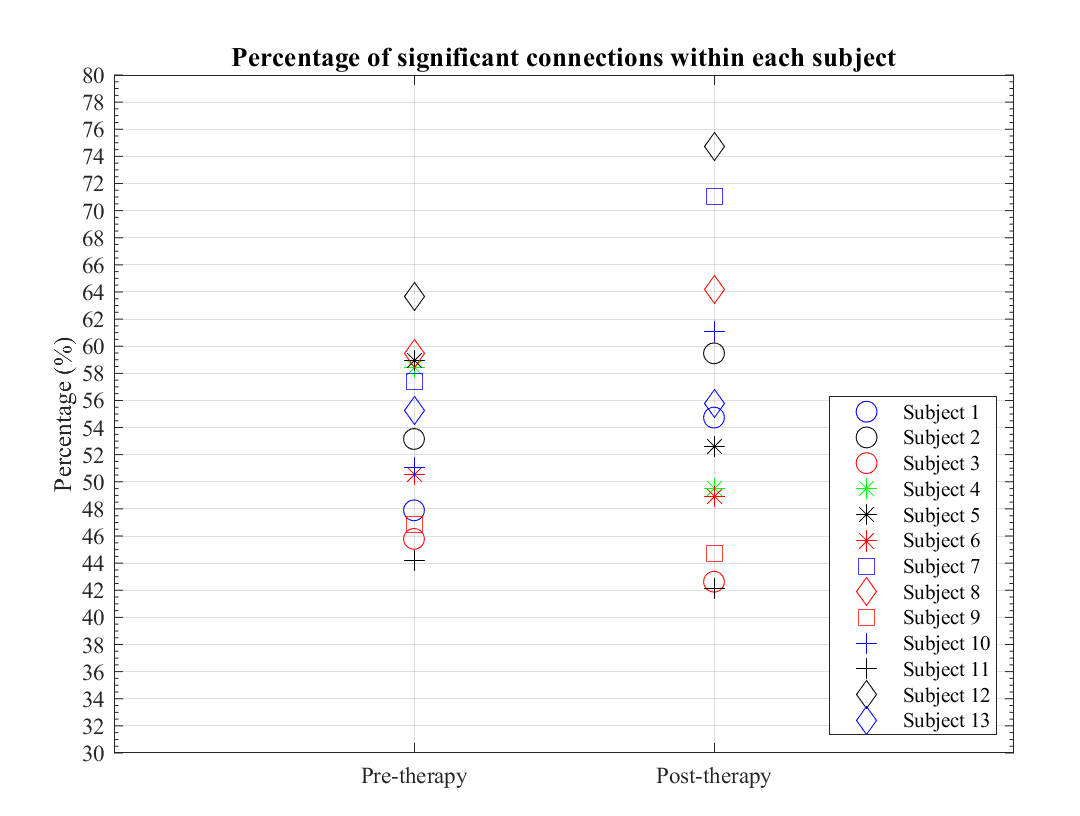

Supplement: Supplementary Figure 1 — Percentage of significant FCs for each patient in each session. Percentage of significant is defined as the number of significant FCs divided by the number of all possible FCs in the network. [file Image_1.TIF]
